# Prehospital tranexamic acid decreases early mortality in trauma patients: a systematic review and meta-analysis

**DOI:** 10.3389/fmed.2025.1552271

**Published:** 2025-03-14

**Authors:** Yi Li, Mei Tian, Wen Zhong, Jiatong Zou, Xin Duan, Haibo Si

**Affiliations:** ^1^Department of Orthopedics and Orthopedic Research Institute, West China Hospital, Sichuan University, Chengdu, China; ^2^Department of Ultrasonography, West China Hospital, Sichuan University, Chengdu, China; ^3^Department of Emergency, West China Hospital, Sichuan University, Chengdu, China

**Keywords:** tranexamic acid, trauma, prehospital application, mortality, adverse outcome

## Abstract

**Background:**

As an anti-fibrinolytic agent, tranexamic acid (TXA) is widely recognized for its efficacy in managing hemorrhagic conditions. Prehospital application of TXA has been reported in recent years, but its benefits in trauma patients remain debated.

**Materials and methods:**

A literature search was conducted across databases including PubMed, Cochrane Library, Embase, Web of Science, SCOPUS, and the Cochrane Central Register for Clinical Trials from inception to October 2024, focusing on studies related to prehospital TXA and clinical outcomes in trauma patients. The Cochrane Risk of Bias 2 Tool was applied to assess the quality of randomized control trials (RCTs), while the Newcastle-Ottawa Scale was used for observational cohort studies. Data were pooled under a random- or fixed-effects model using RevMan 5.4 with odds ratio (OR) and 95% confidence interval (CI) as the effect measures.

**Results:**

A total of 286 publications were identified from the initial database search, and 12 studies, including five RCTs and seven observational cohort studies with a total of 12,682 patients, were included. Significant early survival benefits were observed in patients receiving prehospital TXA compared to those not receiving prehospital treatment. Compared to the control group, the prehospital TXA group exhibited a significant reduction in 24-h mortality with an OR of 0.72 and a 95% CI of 0.54–0.94 (*p* = 0.02), while no statistically significant difference in the incidence of venous thromboembolism (VTE; OR: 1.14, 95% CI: 0.98–1.33, *p* = 0.09). No significant differences were observed in other outcomes, such as 28–30-day mortality, overall mortality, length of hospital stay, and the incidence of multiple organ failure (all *p* > 0.05).

**Conclusion:**

Prehospital TXA decreases early (24-h) mortality in trauma patients without a significant increase in the risk of VTE and other complications, and further studies are still needed to improve and optimize its management strategy.

**Systematic review registration:**

https://www.crd.york.ac.uk/PROSPERO/, Identifier: CRD 42019132189.

## Highlights

- Prehospital TXA decreases early mortality in trauma patients.
- Prehospital TXA does not increase the incidence of venous thromboembolism in trauma patients.
- Prehospital TXA does not increase the incidence of other complications, such as length of hospital stay and multiple organ failure, in trauma patients.

## 1 Introduction

In 2021, approximately 8% of the global mortality rate (4.4 million) was injury-related (1). Trauma remains a leading cause of mortality and disability worldwide, especially within the first 24 h of injury (2, 3). Hemorrhagic shock, especially in the early post-injury period, is the primary cause of death among trauma patients, responsible for 30 to 40% of all trauma-induced fatalities (2, 4). Acute blood loss not only directly leads to mortality but also significantly increases the risk of numerous post-traumatic complications. In response to severe hemorrhage, standard in-hospital management often involves massive transfusion protocols and the administration of hemostatic agents. However, patients with severe traumatic injuries, particularly those with underlying medical conditions, frequently face challenges in stabilizing hemodynamics and mitigating adverse outcomes upon hospital admission. Moreover, prehospital care presents unique challenges in trauma management. Factors such as unidentified bleeding sources, the inability to perform timely cross-matching, and inappropriate prehospital interventions (e.g., excessive fluid resuscitation or the use of medications that may exacerbate hemorrhage) can further complicate patient conditions. These limitations contribute to increased treatment complexity, a higher economic burden, and an elevated risk of adverse outcomes and mortality.

Timely and effective prehospital fluid resuscitation and hemostatic interventions play a critical role in reducing adverse outcomes among trauma patients. Hemostatic resuscitation, primarily transfusion-based, has become the emerging standard of care for hemorrhagic shock in the prehospital setting (5). However, the unpredictability of traumatic events, uncertainty surrounding transfusion indications, and safety concerns regarding emergency blood transfusions limit the practical application of blood products before hospital arrival (6–8). In recent years, increasing attention has been given to the early use of hemostatic agents as an alternative to transfusions, with evidence suggesting their ability to rapidly control bleeding and improving outcomes (9). One such agent, tranexamic acid (TXA), a synthetic lysine analog that inhibits the conversion of plasminogen to plasmin, has been widely investigated since its synthesis in 1962. By preventing fibrinolysis and clot degradation, TXA reduces blood loss and has demonstrated significant benefits in patients with hemorrhagic shock (10).

The Clinical Randomization of an Anti-fibrinolytic in Significant Hemorrhage 2 (CRASH-2) trial, for example, revealed that early administration of TXA (within 3 h of injury) in adults with significant hemorrhage or at risk of hemorrhage significantly reduced mortality related to hemorrhage (11). These findings have led to the widespread use of TXA in various bleeding conditions, including trauma, postpartum hemorrhage, traumatic brain injury, gastrointestinal bleeding, hemoptysis, thrombolysis-induced major bleeding, and surgical bleeding (12, 13). Despite growing evidence supporting the early prehospital TXA in trauma patients, there remains considerable debate (14, 15). For example, the timing of TXA administration can differentially impact patient outcomes. Moreover, concerns have been raised regarding the potential for TXA to tip the balance between thrombogenesis and thrombolysis, leading to an increased risk of thromboembolic events, such as venous thromboembolism (VTE) or stroke (16). Given these conflicting perspectives, it is crucial to undertake a comprehensive, evidence-based review to evaluate the efficacy and safety of TXA in prehospital blood management for trauma patients.

This study conducted a systematic review and meta-analysis of existing literature on the prehospital TXA in trauma patients. As a result, our findings indicate that prehospital TXA significantly decreases early (24-h) mortality in trauma patients, providing essential insights for optimizing prehospital blood management strategies involving TXA. Future research should focus on identifying the optimal timing and dosage of TXA administration and determining which subsets of trauma patients are most likely to benefit from prehospital TXA.

## 2 Materials and methods

**Figure 1:**
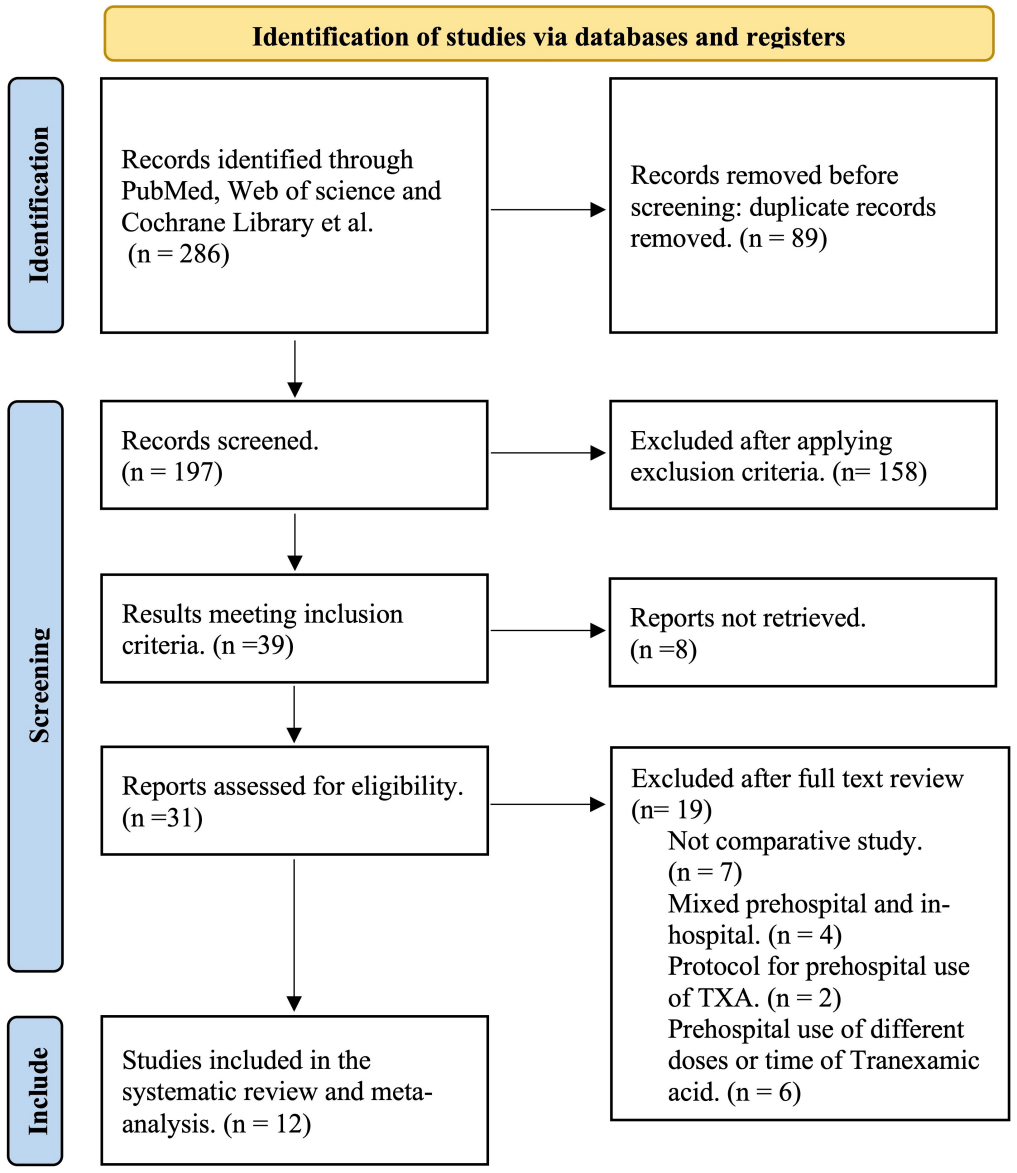
Flow diagram of the study selection process.

The systematic review and meta-analysis were carried out following the Cochrane Guidance for Systematic Review and Meta-Analysis and adhered to the Preferred Reporting Items for Systematic Reviews and Meta-analysis (PRISMA) guidelines (17, 18). This study was registered in the International Prospective Register of Systematic Reviews. Quality assessments were conducted using the AMSTAR 2 checklist (Supplementary document 1) (19). The flowchart outlining the study selection process is presented in Figure 1.

### 2.1 Inclusion/exclusion criteria

The PICOS principle was used to formulate the inclusion criteria.

Population: adult patients with trauma-induced known or suspected hemorrhage. Suspected hemorrhage refers to clinical signs that suggest blood loss without definitive confirmation.

Intervention: prehospital administration of TXA.

Comparison: no administration of prehospital TXA.

Outcomes: the primary outcome was mortality, measured within 24 h and at 28 to 30 days, and the incidence of VTE, including pulmonary embolism and deep vein thrombosis. The secondary outcome was the length of hospital stay (LOHS), incidence of multiple organ failure (MOF), and overall mortality. All outcomes were recorded during the follow-up period.

Study design: randomized control trials (RCTs), case-control studies, or cohort studies published in English.

Exclusion criteria: We excluded narrative reviews, protocols, unpublished reports, editorials, clinical case reports, commentaries, and abstracts as they did not align with the focus of our study. We excluded the population under 18 years, studies not focusing on prehospital TXA, and articles not published in English. This study did not exclude patients on anticoagulants, previous cardiac events, hypertensive patients on medication, or those with other chronic illnesses who sustained trauma.

### 2.2 Search strategy

A systematic search was conducted using Medline (PubMed), Cochrane Library, Embase, Web of Science, SCOPUS, and the Cochrane Central Register for Clinical Trials from inception until October 2024. A gray literature search was performed and focused on the following databases: World Health Organization, International Clinical Trial Registry Platform, Clinicaltrials.gov, European Clinical Trial, Registry, Google search, and Google Scholar. The search strategy combined keywords, Mesh terms, and synonyms pertinent to “prehospital,” “Out-of-Hospital,” “tranexamic acid,” “trauma,” “hemorrhage,” and “coagulation.” Searches of keywords and terms were initially expanded with the Boolean Operator OR. Then, the results of these searches were combined with the Boolean Operator AND. Two independent reviewers screened the search results and applied eligibility criteria to identify potentially relevant articles.

### 2.3 Data extraction

The research discussion and conclusion were considered from the perspective of demonstrating the impact of prehospital TXA on mortality and other adverse outcomes. Hence, the following data were extracted from each article: publication date, number of participants, study design, follow-up period, study base (civilian or military), region, analysis methods, timing and location of the first TXA administration, and clinical outcome measures. Two independent reviewers extracted the data and verified the results simultaneously. In instances of discrepancies or divergences in opinion between the two primary researchers, a third senior researcher was consulted to arbitrate and provide a definitive inclusion decision.

### 2.4 Ethical approval

This study reviews data from previously published studies without collecting any new data from patients directly, and ethical approval was not required.

### 2.5 Quality assessment of the studies and risk of bias

**Figure 2:**
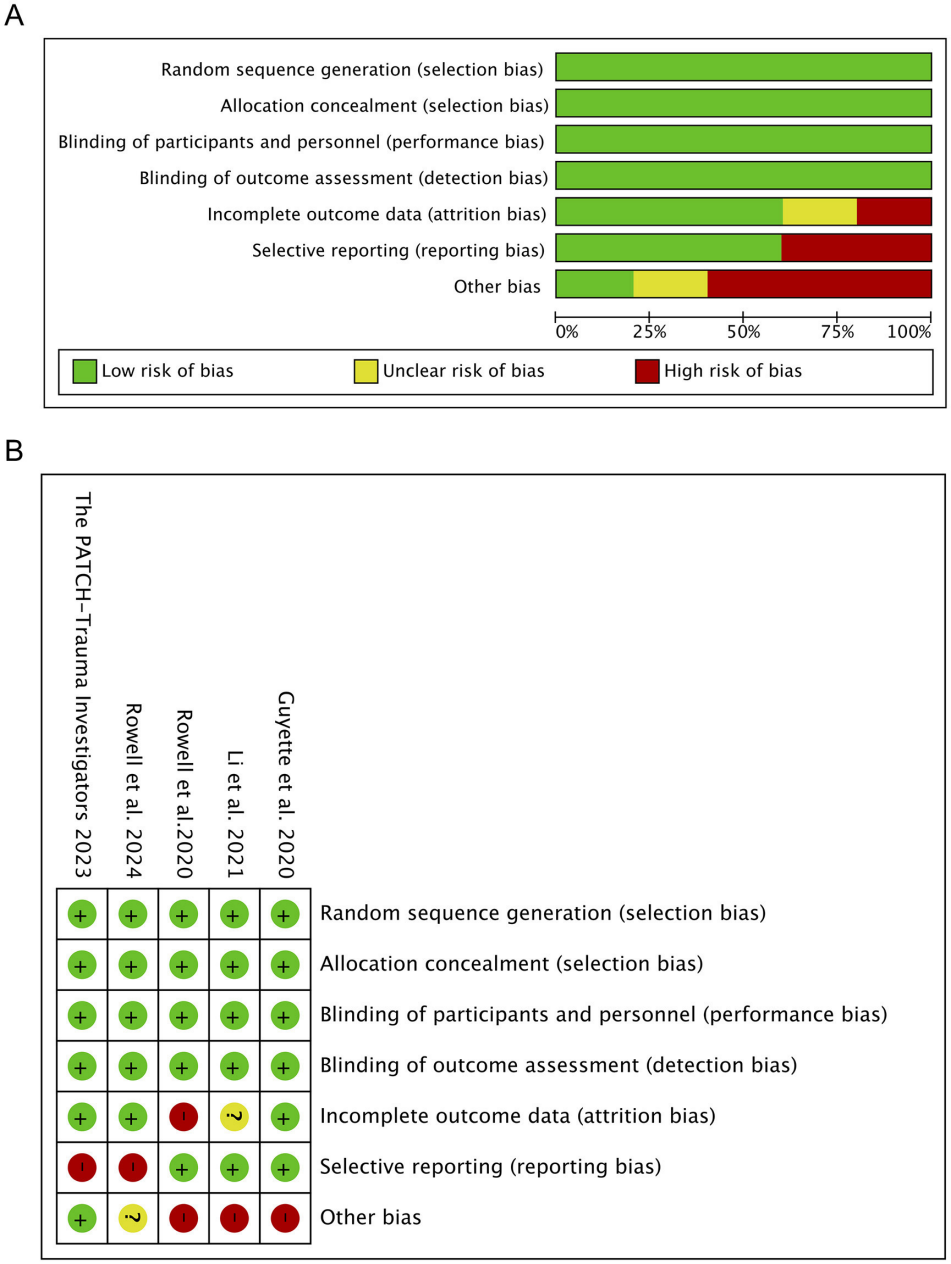
Quality assessment of included trials. **(A)** Risk of bias graph. **(A)** Shows the proportions of various bias risks, such as random sequence generation and allocation concealment, in a bar chart. **(B)** Risk of bias summary. **(B)** Summarize the specific results of each bias risk in each study, in tabular form.

The quality and risk of bias for RCTs were assessed using the Cochrane Risk of Bias 2 (RoB 2) Tool, and each domain was classified into low, unclear, and high risk based on different bias risks to ensure study reliability (20). Non-randomized studies were evaluated using the Newcastle-Ottawa Scale (NOS), with a maximum score of nine points (21). We applied the NOS to assess their quality based on three domains: selection, comparability, and outcome measurement. High-quality studies score 7–9, meeting most criteria across domains; moderate-quality studies score 4–6, meeting some criteria but with flaws; low-quality studies score 0–3, failing to meet several criteria. Two independent reviewers conducted these assessments, with consideration of a third reviewer in case of discrepancy or conflict. Adopting the evidence grading and recommendation intensity standards developed by the Evidence-Based Medicine Center at the University of Oxford in the UK to rate the included literature into a level of evidence, ranging from 1a (strong evidence) to five (weak evidence). The results of the risk of bias assessments are presented in Figure 2 and Table 1.

**Table 1:** Baseline characteristics, primary outcomes, level of evidence, NOS scores and results of included studies.

**Author**	**Study period**	**Country**	**Study design**	**Propensity score analysis**	**Patients (n)**		**Ages [years, mean (SD)]**		**Male/Female (n)**		**Follow-up**	**Primary outcomes**	**Level of evidence**	**NOS scores**	Results^*^
					**PH TXA**	**NO TXA**	**PH TXA**	**NO TXA**	**PH TXA**	**NO TXA**					
Wafaisade (29)	2012–2014	Germany	Multicenter prospective cohort study	Yes	258	258	43.00 (19.00)	41.00 (18.00)	187/71	187/71	30 days	Mortality (24 h and 30 d), VTE	2a	7	+
El-Menyar (32)	2017–2018	Qatar	Single center retrospective cohort study	No	102	102	31.41 (0.90)	31.49 (0.90)	98/4	98/4	Unknown	Mortality (unknown time), VTE	2b	7	–
Van Wessem (52)	2013–2021	Netherlands	Single center prospective cohort study	No	120	114	41.30 (27.01)	50.19 (24.03)	80/40	77/37	28 days	Mortality (unknown time), VTE	2b	6	–
Imach (33)	2015–2019	Germany	Multicenter retrospective cohort study	Yes	2,275	2,275	47.6 (19.9)	47.5 (19.9)	1,679/596	1,688/587	30 days	Mortality (6 h, 12 h, 24 h and 30 day), VTE	2a	7	+
Neeki (31)	2015–2017	USA	Multicenter prospective cohort study	Yes	362	362	37.96 (16.11)	37.64 (16.33)	293/69	293/69	28 days	Mortality (24 h, 48 h and 28 day), VTE	2a	7	+
Bossers (15)	2012–2017	Netherlands	Multicenter prospective cohort study	No	693	1,134	45.95 (30.46)	43.95 (31.92)	486/207	797/337	1 year	Mortality (30 day)	3a	7	–
Max Gulickx (28)	2015–2017	Netherlands	Multicenter retrospective cohort study	No	124	353	38.49 (21.45)	42.81 (25.38)	97/27	238/115	30 days	Mortality (24 h and 30 day)	1b	7	±
Li et al. (24)	2022	USA	Multicenter RCT	No	244	232	39.75 (21.63)	40.75 (23.12)	167/77	165/67	30 days	Mortality (30 day), VTE	1b	–	+
Guyette (23)	2015–2019	USA	Multicenter RCT	No	447	456	41.00 (17.00)	42.00 (18.00)	327/120	341/115	30 days	Mortality (24 h and 30 day), VTE	1b	–	±
Rowell (25)	2015–2017	USA, CAN	Multicenter RCT	No	657	309	40.72 (22.64)	38.81 (22.34)	482/175	233/76	6 months	Mortality (28 day), VTE	1b	–	±
PATCH-Trauma Investigators and the ANZICS Clinical Trials Group (26)	2014–2021	Australia, New Zealand, and Germany	Multicenter RCT	No	657	643	44.10 (19.70)	44.20 (18.90)	459/198	459/1,184	6 months	Mortality (24 h and 28 day), VTE	1b	–	±
Rowell et al. (27)	2015–2017	USA, CAN	Multicenter RCT	No	370	171	43.53 (19.46)	42.00 (19.00)	266/104	136/35	6 months	Mortality (24 h and 28 day), VTE	1b	–	±

NOS, Newcastle-Ottawa Scale; SD, Standard deviation.

+ positive correlation between PH TXA and outcomes; – negative correlation between PH TXA and outcomes; ± both positive and negative correlation between PH TXA and outcomes were reported.

### 2.6 Data analysis

To evaluate the effect of TXA on outcomes, odds ratio (OR) with a 95% confidence interval (CI) and *p*-value were calculated for mortality at 24 h and 28 to 30 days and overall mortality during the follow-up period, incidence of VTE, MOF, and LOHS. The forest plot was generated using Cochrane Review Manager (RevMan, version 5.4). Heterogeneity among studies was assessed using the I square (*I*^2^) statistic, with thresholds of 0–25% indicating low heterogeneity, 25–50% moderate heterogeneity, 50–75% high heterogeneity, and >75% considerable heterogeneity (22). For *I*^2^ values >50%, an M-H random-effects model was used; otherwise, an M-H fixed-effects model was selected. Publication bias was evaluated visually by funnel plots and statistically with Egger's regression test for outcomes with 10 or more studies. Due to the limited number of studies, funnel plots were not presented. A *p* < 0.05 was considered a statistically significant publication bias.

## 3 Result

### 3.1 Selection and risk assessment of the studies

The initial database search identified 286 results. After removing duplicates, the search identified 197 unique citations, of which 39 met the inclusion criteria. Eight results were not retrieved, and 19 studies were excluded due to the absence of comparative data, mixed prehospital and in-hospital use of TXA, or protocols for prehospital TXA. Ultimately, 12 studies were included in the review, comprising five multi-centered RCTs (23–27), five multi-centered cohort studies (16, 28–31), and two single-center cohort studies (32, 33). The baseline characteristics and outcomes of the included studies are summarized in Table 1. The studies were conducted over a 1-year to 5-year period, spanned from 2012 to 2024, and were conducted in the following geographical areas: the United States, Canada, Germany, Qatar, Australia, New Zealand, and the Netherlands. A total of 12,682 patients were included, with no significant differences in patient numbers between groups in the individual studies. Five studies reported 30-day outcomes, four studies had 28-day follow-up reports, and one study did not specify the follow-up duration. Mortality time points were variably reported as 6 h, 12 h, 24 h, 48 h, 28 days, 30 days, and 6 months. Nine studies reported VTE events.

### 3.2 Quality and risk of bias assessment

The quality of RCTs was evaluated using the Cochrane risk of bias assessment tool. According to the random allocation method, concealment of the allocation scheme, blinding of subjects, blinding of outcome assessment, data integrity, selective reporting, and other sources of bias, the quality of different studies was divided into high-risk, low-risk, and unclear. The mapping was completed by Review Manager 5.4 software, and the results are presented in Supplementary Table 1. Cohort studies were evaluated using the Newcastle-Ottawa Scale (NOS), which scores studies based on the selection of subjects in different cohort studies, comparability between groups, and outcome measurement (Supplementary Table 2). The maximum score is nine, with the final score for each study detailed in Table 1. Based on these quality evaluations and risk of bias assessments, the level of evidence ranged from 1a (strong evidence) to five (weak evidence).

### 3.3 Results of meta-analysis

#### 3.3.1 Impact of prehospital TXA on 24-h mortality

**Figure 3:**
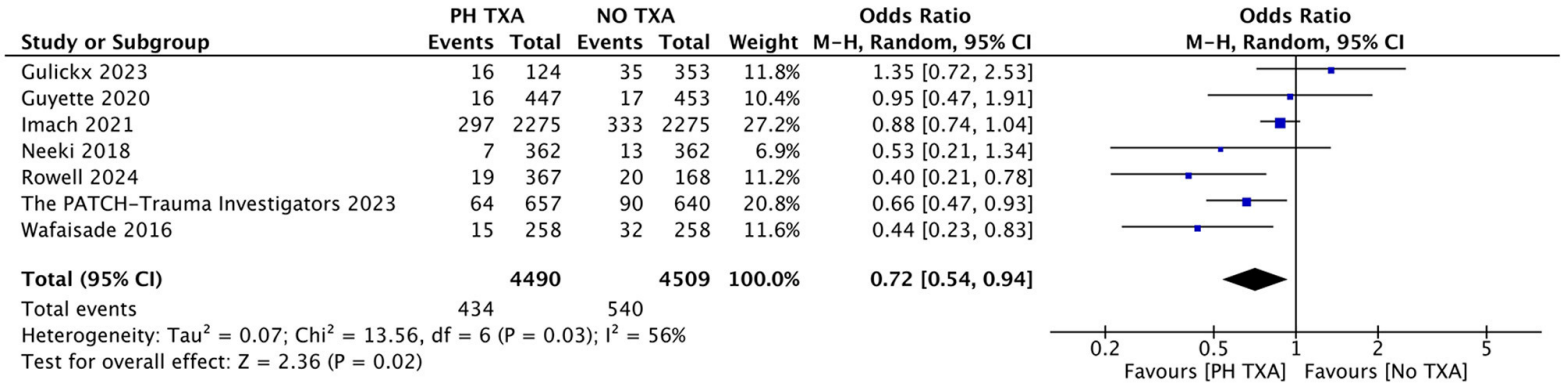
Forest plot of the effect (OR with 95% CI) of prehospital TXA on 24-h mortality. The results suggest that prehospital use of TXA may be associated with a lower 24-h mortality rate.

Seven studies reported 24-h mortality (Figure 3) (16, 23, 27–29, 31, 33). The prehospital TXA (PH TXA) group consisted of 4,490 patients, while the no TXA (NO TXA) group included 4,509 patients. There was a 19.6% reduction in deaths among PH TXA (*n* = 434) compared to NO TXA (*n* = 540) patients (OR, 0.72; 95% CI, 0.54–0.94; *p* = 0.02).

#### 3.3.2 Impact of prehospital TXA on the risk of VTE

**Figure 4:**
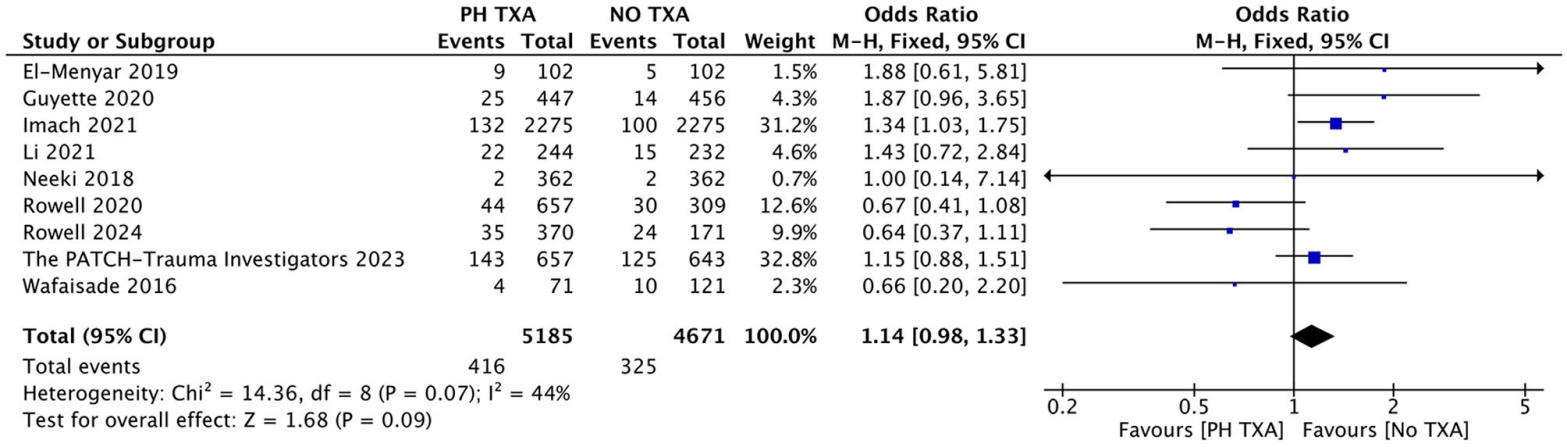
Forest plot of the effect (odds ratio with 95% confidence interval) of prehospital TXA on VTE development. The results show that there is no significant difference in the incidence of VTE between prehospital use of TXA and non-use.

Nine studies reported the incidence of VTE (16, 23–25, 27, 29, 31–33). The PH TXA group included 5,185 patients, and the NO TXA group had 4,671 patients (Figure 4). There was a 28.0% increase in VTE among PH TXA patients (*n* = 416) compared to NO TXA patients (*n* = 325), but this increase did not reach statistical significance (OR, 1.14; 95% CI, 0.98–1.33; *p* = 0.09).

#### 3.3.3 Impact of prehospital TXA on 28–30-day mortality

**Figure 5:**
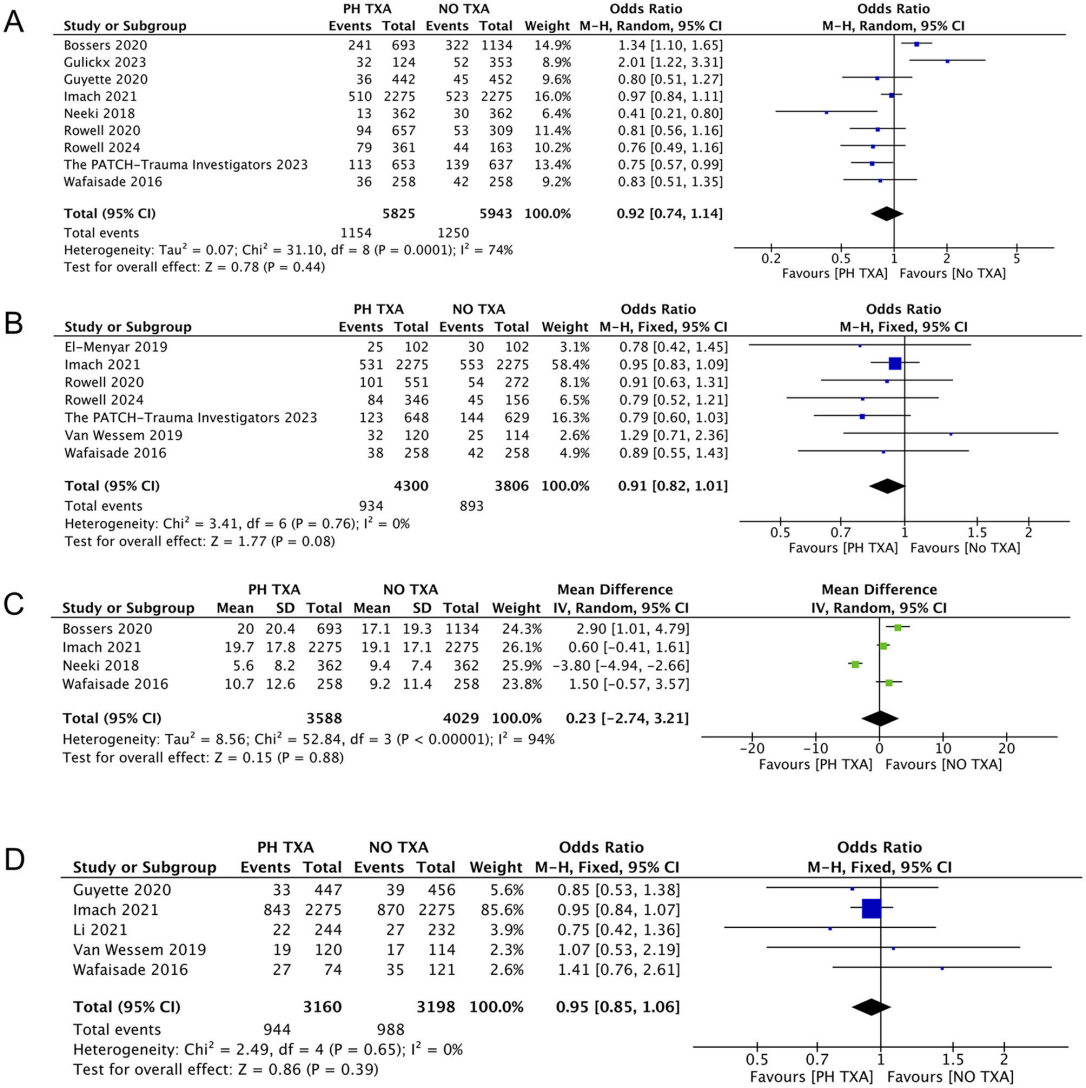
Forest plot of other outcomes. **(A)** Forest plot of the effect (odds ratio with 95% confidence intervals) of prehospital TXA on 28 to 30 days mortality. **(B)** Forest plot of the effect (odds ratio with 95% confidence intervals) of prehospital TXA on overall mortality. **(C)** Forest plot of the effect (odds ratio with 95% confidence intervals) of prehospital TXA on LOHS. **(D)** Forest plot of the effect (odds ratio with 95% confidence intervals) of prehospital TXA on MOF. The results show that there is no significant difference in the incidence of 28 to 30 days mortality, overall mortality, LOHS and MOF between prehospital use of TXA and non-use.

Nine studies reported mortality, including five reported 30-day mortality and four reported 28-day mortality (Figure 5) (15, 16, 23, 25, 27–29, 31, 33). The PH TXA group comprised 5,825 patients, while the NO TXA group included 5,943 patients. There was a trend toward reduced mortality in the PH TXA group (*n* = 1,154) compared to the NO TXA group (*n* = 1,250), though this did not reach statistical significance (OR, 0.92; 95% CI, 0.74–1.14; *p* = 0.44).

#### 3.3.4 Impact of prehospital TXA on overall mortality

Seven studies reported overall mortality during the follow-up period (16, 25–27, 29, 32, 33). The PH TXA group comprised 4,300 patients, and the NO TXA group had 3,806 patients (Figure 5). There was a trend toward reduced overall mortality in the PH TXA group compared to the NO TXA group, but this was not statistically significant (OR, 0.91; 95% CI, 0.82–1.01; *p* = 0.08).

#### 3.3.5 Impact of prehospital TXA on LOHS

Four studies reported data on LOHS (15, 29, 31, 33). The PH TXA group had 3,588 patients, and the NO TXA group had 4,029 patients (Figure 5). There was no statistical significance in LOHS between the PH TXA and NO TXA groups (OR, 0.23; 95% CI, −2.74–3.21; *p* = 0.88).

#### 3.3.6 Impact of prehospital TXA on MOF

Five studies reported MOF (16, 24, 26, 29, 33). The PH TXA group included 3,160 patients, and the NO TXA had 3,198 patients (Figure 5). There was a trend toward reduced MOF in the PH TXA group compared to the NO TXA group, but this was not statistically significant (OR, 0.95; 95% CI, 0.85–1.06; *p* = 0.39).

### 3.4 Systematic review

Hemodynamic stability plays a vital role in the management of patients with severe trauma. Blood loss may lead to end-organ damage, and current guidelines recommend early administration of TXA to control bleeding (26). TXA exhibits prothrombotic effects and may pose potential adverse myocardial effects, and a monocentric cohort study investigated the association between TXA use and myocardial injury in patients with severe trauma (34). The study included 297 patients aged ≥18 with an injury severity score (ISS) of ≥16, treated at a tertiary hospital from 2016 to 2019. Multivariate logistic regression models indicated that the OR values for prehospital TXA with myocardial injury, major adverse cardiovascular events (MACE), and mortality were 0.75 (95% CI, 0.25–2.23), 0.51 (95% CI, 0.06–4.30), and 0.84 (95% CI, 0.21–3.33], respectively. This study showed that early administration of TXA was not associated with myocardial injury or increased MACE incidence, emphasizing the cardiovascular safety of early TXA use in trauma settings. Another RCT investigated the timing of prehospital TXA on outcomes after traumatic brain injury (TBI). Among patients with moderate to severe TBI, no mortality benefit was observed when TXA was administrated within 45 min compared to within 2 h of injury, despite a low incidence of selective complications (35). These results support early TXA administration (within 45 min) in patients with suspected TBI (36).

For injured patients at risk of bleeding, prehospital packed red blood cells (PRBC) combined with TXA was associated with reduced 30-day mortality, while PRBC transfusion alone reduced early mortality (37). Potential synergies only emerged in longer-term mortality. Prehospital professionals equipped to administer PRBC and TXA should consider TXA for any patient meeting prehospital PRBC transfusion criteria. Further research is needed to elucidate the mechanisms underlying this therapeutic benefit and optimize prehospital resuscitation strategies. Additionally, prehospital TXA may reduce the damage and dysregulation of downstream endothelium and the immune system (38). A secondary analysis of a prospective randomized trial indicated that prehospital TXA was associated with lower levels of syndecan-1 upon admission, suggesting that TXA may mitigate endothelial injury post-trauma (39).

### 3.5 Publication bias

The inverted or asymmetric funnel plot suggests a potential risk of publication bias due to the limited number of included studies.

## 4 Discussion

TXA is a synthetic hemostatic agent that inhibits fibrinolysis and has been shown to effectively reduce bleeding and mortality in trauma, peripartum, and perioperative settings (36). In recent years, prehospital TXA in trauma patients has gathered attention, though its benefits and potential adverse reactions remain subjects of debate. This systematic review and meta-analysis synthesized the current evidence on prehospital TXA administration in trauma patients, and the findings indicate that prehospital TXA significantly reduces early (24-h) mortality without a significant increase in the risk of thromboembolism. Meanwhile, no significant differences were observed in other outcomes, such as 28–30-day mortality, overall mortality, LOHS, and the incidence of MOF. Therefore, prehospital TXA demonstrates a clear advantage in reducing early mortality among trauma patients, although further research is necessary to strengthen the clinical applicability and explore the potential risks.

Hemorrhage due to trauma is one of the leading causes of morbidity and mortality, with uncontrolled prehospital bleeding being the primary contributor to death in hemorrhagic trauma patients. Early and aggressive intervention is critical to preventing further complications (8, 40). Currently, the cornerstone of hemorrhage control resuscitation is the early and appropriate use of blood products and adjuncts, such as the infusion of component or whole blood, based on the recognition of trauma-induced coagulopathy and the dilutional coagulopathy during crystalloid resuscitation. Moreover, pharmacological agents that enhance hemostasis are a growing area of research (41, 42). However, given the sudden onset of prehospital trauma, the limited availability of blood products, and the potential adverse effects of uncross-matched blood transfusions, hemostatic drugs like TXA offer distinct advantages. TXA facilitates bleeding control in prehospital settings with fewer adverse reactions, making it a practical choice for trauma patients before hospital arrival (43, 44).

Early hemostasis is crucial for preventing and managing adverse outcomes in hemorrhagic diseases (45). Abdulrahman et al. reported that the mortality risk for patients receiving TXA before hospitalization is significantly lower than that of the control group, with a lower incidence of thromboembolic events in the TXA group (46). In this meta-analysis, the prehospital TXA was associated with a significant reduction in early mortality and a decreasing trend in overall mortality compared to patients who did not receive TXA, along with previous studies that also demonstrated a reduction in early mortality (22, 46). Earlier studies were often limited by small samples, while this study includes larger sample sizes and more comprehensive literature, providing a significant update and strengthening the evidence for TXA's efficacy in reducing early mortality. Therefore, the early prehospital of TXA is confirmed as an effective intervention for controlling hemorrhage and managing blood loss in trauma patients, supporting its inclusion as a reliable prehospital treatment strategy.

Although prehospital TXA is associated with a reduced need for blood product transfusions in the hospital (47), it does not offer an advantage regarding LOHS or intensive care unit (ICU) stay, according to our meta-analysis. Some studies recommend TXA use for severely injured patients who require blood transfusion and also emphasize the need to monitor for VTE due to the increase in D-dimer levels, a known risk factor for VTE (46). In the subacute stage of chronic inflammation or trauma, the elevation of coagulation factors such as factor VIII/von Willebrand factor (VWF) can enhance thrombin production and promote thrombosis by augmenting inflammatory responses (48). Trauma patients under stress may trigger the body's anticoagulation system, increasing the likelihood of thrombus formation (49). Despite these concerns, no research has specifically addressed the mortality rate caused by VTE following prehospital TXA. Importantly, the potential increase in VTE risk must be weighed against the critical benefit of promoting early survival.

A consensus has emerged on the early and extensive use of TXA, carbazole sulfonate sodium, pituitrin, and other hemostatic agents in managing hemorrhagic disease. However, a significant complication associated with these interventions is the risk of severe or life-threatening thromboembolism (50, 51). The relationship between effective hemostasis and the risk of thromboembolism is complex, and striking an optimal balance between efficacy and safety remains a critical concern when using hemostatic drugs. This meta-analysis revealed that prehospital TXA was associated with an increasing trend in the risk of VTE, even though it was not statistically significant. Among the nine studies included, only one reported a significant increase in VTE events, while the remaining studies found no significant difference in VTE risk between the TXA and control groups (23). Additionally, our study did not exclude patients on anticoagulants, those with previous cardiac events, hypertensive patients on medication, or individuals with other chronic illnesses sustained trauma, which could affect outcomes, particularly in terms of delayed mortality. In the future, multi-center, high-quality RCTs with larger sample sizes are urgently needed to validate the findings and clarify the VTE risk associated with prehospital TXA.

This meta-analysis found no significant differences in other observed outcomes, such as 28–30-day mortality or the incidence of MOF. Similarly, the CRASH-2 trial reported improved 28-day survival and reduced bleeding-related deaths, with no significant difference in VTE rates between TXA and placebo groups (10). Among the studies included in this analysis, only one examined the relationship between prehospital TXA and blood transfusion requirements (22). Regardless of the volume of blood transfused in the first 4 h, total in-hospital blood transfusion, or the number of patients requiring massive blood transfusion (defined as ten or more units of red blood cells within 24 h or more than 40 ml/kg PRBC in 2 h or less) was significantly higher in the placebo group than in the TXA group, suggesting that prehospital TXA is associated with a significant reduction in transfusion requirements (22). Other potential outcome measures, such as the risk of perioperative infections, effects on blood glucose and blood pressure, functional recovery after injury, and long-term prognosis following prehospital TXA, warrant further investigation to understand the broader implications of TXA in trauma care.

This study also has several limitations. Firstly, among the studies included in this meta-analysis, some had disproportionate influence due to their large sample sizes (33), which may skew the overall results. Secondly, no study specifically reported the relationship between prehospital TXA and blood loss, making it impossible to assess whether TXA reduces blood loss in prehospital settings. Meanwhile, the lack of studies on the dose, duration, and mode of administration of prehospital TXA also hinders our understanding of these variable's impact on trauma outcomes such as thromboembolism. Thirdly, this study only focused on the administration of TXA before hospital arrival and did not account for the potential impact of concurrent use of blood transfusions or substitutes, vasoactive drugs, or fluid resuscitation on the outcomes. Fourthly, when evaluating the treatment outcomes of trauma patients, several critical factors need to be considered, such as pre-existing vascular malformations, comorbidities, daily medications administered, and patients' adherence to the treatment regimen. These factors may potentially impact the overall treatment process and its outcomes. Meanwhile, the included studies have not yet explored the correlation between the timing of administering TXA as a medical intervention and its potential side effects on both short-term and long-term outcomes. Lastly, this review was restricted to English-language publications, possibly omitting relevant studies in other languages. Therefore, as an emerging and promising area, the prehospital TXA needs further comprehensive investigation and elucidation in numerous aspects.

## 5 Conclusions

In conclusion, prehospital TXA significantly decreases early (24-h) mortality in trauma patients without a significant increase in the risk of thromboembolism and other complications, highlighting the potential life-saving benefits of prehospital TXA in patients with hemorrhagic conditions. Nevertheless, as an emerging and promising area, the prehospital TXA requires further comprehensive investigation and elucidation to improve and optimize its management strategy, and future research should focus on optimizing the timing, dosage, frequency, concurrent use with other agents, and strategies for preventing and managing complications.

## Data Availability

The original contributions presented in the study are included in the article/Supplementary material, further inquiries can be directed to the corresponding author.
