# Supplementary material for: Prehospital tranexamic acid decreases early mortality in trauma patients: a systematic review and meta-analysis
Source: Front Med (Lausanne). 2025 Mar 14;12:1552271. doi: 10.3389/fmed.2025.1552271 (PMC11951308; doi:10.3389/fmed.2025.1552271)
Supplement: Supplementary file 1 [file Data_Sheet_1.DOCX]

**Supplemental Table 1**

Cochrane risk of bias assessment for the Randomized Control Trials.

| **Author** | **Selection bias** | | **Performance bias** | **Detection bias** | **Attrition bias** | **Reporting bias** | **Other bias** |
| --- | --- | --- | --- | --- | --- | --- | --- |
|  | Random sequence generation | Allocation concealment | Blinding of participants and personnel | Blinding of outcome assessment | Incomplete outcome data | Selective reporting | Grade level |
| Guyette 2020 | Low | Low | Low | Low | Low | Low | High |
| Rowell 2020 | Low | Low | Low | Low | High | Low | High |
| Li 2021 | Low | Low | Low | Low | Unclear | Low | High |
| The PATCH-Trauma Investigators and the ANZICS Clinical Trials Group 2023 | Low | Low | Low | Low | Low | High | Low |
| Rowell 2024 | Low | Low | Low | Low | Low | High | Unclear |

**Supplemental Table 2**

Newcastle-Ottawa risk of bias and quality assessment for observational studies.

| **Author** | **Selection** | | | |  | **Comparability** | |  | **Outcomes** | | | **Total stars (scores)** |
| --- | --- | --- | --- | --- | --- | --- | --- | --- | --- | --- | --- | --- |
|  | Representative of the intervention cohort | Selection of control cohort | Ascertainment of intervention | Demonstration that outcome of interest was not present at start of study |  | Age, gender, ISS, HR and SBP | Blood transfusion, race and injury category |  | Assessment of outcomes | Adequacy of follow-up of cohorts | Completeness of the intervention versus the control cohort |  |
| Imach 2021 | * | * | * | * |  | * |  |  | * |  | * | ******* (7) |
| Bossers 2020 | * | * | * | * |  | * |  |  | * |  | * | ******* (7) |
| El-Menyar 2019 | * | * | * | * |  | * |  |  | * |  | * | ******* (7) |
| Van Wessem 2019 | * | * | * | * |  | * |  |  | * |  |  | ****** (6) |
| Neeki 2018 | * | * | * | * |  | * |  |  | * |  | * | ******* (7) |
| Wafaisade 2016 | * | * | * | * |  | * |  |  | * |  | * | *******(7) |
| Gulickx 2023 | * | * | * | * |  | * | * |  | * |  | * | ********(8) |
| ***Abbreviations:*** *ISS: injury severity score, HR: heart rate, SBP: systolic blood pressure.* | | | | | | | | | | | | |
